# Determinants of Breast Cancer Screening Practice among Women in Indonesia: A Nationwide Study

**DOI:** 10.31557/APJCP.2021.22.5.1435

**Published:** 2021-05

**Authors:** Solikhah Solikhah, Lianawati Lianawati, Ratu Matahari, Dwi Sarwani Sri Rejeki

**Affiliations:** 1 *Faculty of Public Health, Universitas Ahmad Dahlan, Yogyakarta, Indonesia. *; 2 *Department of Public Health, Faculty of Health Sciences, Universitas Jenderal Soedirman, Purwokerto, Indonesia. *

**Keywords:** Breast cancer, breast cancer screening, knowledge of breast cancer risk factors, Indonesia

## Abstract

**Background:**

Breast cancer remains the leading cause of death for women globally, including in Indonesia. Breast cancer screening plays a vital role in reducing deaths caused by breast cancer. However, breast cancer screening rate is still low and studies on determinants for breast cancer screening is limited in Indonesia. This study aimed to identify the determinants of breast cancer screening among women in Indonesia.

**Methods:**

This population-based study was conducted among 827 women who lived in either rural and urban areas, using a stratified sampling design where were based on province and locality combinations. Data were analysed using a binary logistic regression model to assess the associations between independent and dependent variables.

**Results:**

As many as 827 women with an average age of 29.91 (± 11.14) years old participated in this study. The overall breast cancer screening among women was 18.74%. Knowledge of breast cancer risk factors, signs, and symptoms (adj.OR = 1.75, 95%CI: 1.20 – 2.56), age of 35 to 39 years old (adj.OR. = 1.52, 95% CI: 1.02 – 2.26), and household income of ≥6,000,000 IDR (≥457 USD) (adj.OR. = 5.19, 95%CI: 1.43–18.84) were associated with breast cancer screening attendance. In contrast, Christian women had a significantly lower breast cancer screening rate that women from other religions (adj. OR. = 0.45, 95%CI: 0.24 – 0.85).

**Conclusion:**

The overall breast cancer screening attendance was poor among Indonesian women population. Age, household income, religion, and knowledge of breast cancer risk factors were identified as the determinant factors for breast cancer screening.

## Introduction

Breast cancer is widely known as a global women’s health problem and the leading cause of death (601,000 deaths) among women in all countries in the world, including Indonesia (Fitzmaurice et al., 2019). According to the World Health Organization (WHO) (2016), 73% of all deaths in Indonesia is related to non-communicable diseases (NCDs), in which 12% are assigned to cancer. Most cancer patient deaths are linked to a delayed diagnosis that patients have presented an advanced stage during their first contact with health providers. This is especially true for developing countries like Indonesia. Breast cancer screening is suggested to play a vital role in reducing the mortality rate of breast cancer (Zielonke et al., 2020). Breast cancer in Indonesia ranks first as the cause of death among women in Indonesia. Based on the data from the Indonesian Basic Health Research (2013), the prevalence of cancer among the population of all ages in Indonesia is 1.4%, with the highest in Yogyakarta Province with 4.1%. There are several options available for breast cancer screening, namely, mammography, ultrasonography, clinical breast examination (CBE), and breast self-examination (BSE) (Mandrik et al., 2019). 

A previous study revealed that mammography is now well recognized and recommended for early detection of breast cancer (Oeffinger et al., 2016). Early presentation of breast cancer and access to optimal treatment are the keys to improved survival rates of breast cancer patients (Coleman, 2017). However, access to mammography is often unaffordable in many developing countries such as Indonesia due to financial barriers and limited health care resources (Tabrizi et al., 2018). By contrast, in developed countries, when organized mammography screening-based national population programs conducted in their health care setting, most breast cancer patients improved their prognosis (Siegel et al., 2019). In addition, various socio-economic factors (e.g., access to health insurance) and psychological factors (e.g., fears, embarrassment, pain) also become barriers for accessing breast cancer screening (Abraído-Lanza et al., 2015; Tripathi et al., 2017; Unger-Saldaña et al., 2018). Adequate knowledge of breast cancer signs and symptoms also become one of the factors that correlate with CBE and mammogram utilization in the form of regular breast check-up. 

In Indonesia, a significant number of women are diagnosed for breast cancer in the advanced stage of the disease due to lack of knowledge and awareness, insufficient social support, numerous psychosocial factors, and, often times, the socio-characteristics of breast cancer patients (Setyowibowo et al., 2017; Anwar et al., 2018; Solikhah et al., 2020). A large-scale study in Ghana found that the educational background of a breast cancer patient is significantly associated with breast cancer screening (Agyemang et al., 2020). Also, many previous studies in both developed and developing countries have demonstrated the followings as factors influencing the breast cancer screening practice, namely: individual and sociodemographic factors; limited access of health insurance; and ethnicity. These factors contribute to the delay in early detection of breast cancer signs and symptoms (e.g., breast swelling, discharge their nipple, pain of armpit, etc.) (Ghanbari et al., 2020; Wu et al., 2019; Elewonibi and BeLue, 2019; Kosog et al., 2020; Orji et al., 2020). 

To date, there has not been any study that focuses on the determinant factors of breast cancer screening in Indonesian population that they remain largely unexplored despite the fact that the Indonesian Ministry of Health has launched a prevention and early detection program that encourage women to have regular breast check-up using by accessing CBE at the primary health care centers. For that reason, this study aimed to comprehensively identify the determinant factors associated with routine breast cancer screening attendance among Indonesian women. 

## Materials and Methods

*Study design and sample population*

**Figure 1:**
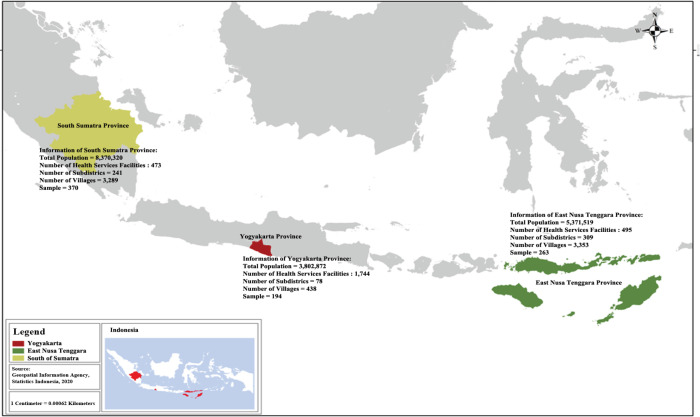
The Sample Areas of This Study Consist of Yogyakarta, South Sumatera and East Nusa Tenggara provinces in Indonesia

This cross-sectional study was conducted in three provinces in Indonesia (South Sumatera, Yogyakarta, and East Nusa Tenggara) that represented the spectrums of heterogeneity in Indonesian culture, religion, and socioeconomic status women. These three provinces were selected purposively as the location of the study because they were included in Indonesia’s effort to prevent breast cancer and able to represent the diverse socioeconomic and culture of Indonesia (Figure 1). All participants living in either rural and urban areas were recruited in this study based on the result of sampling using the stratified sampling method where strata were based on province and locality (rural and urban) combinations frame. The inclusion criteria for this study were Indonesian women aged ≥18 years old living in a rural or urban area of Yogyakarta, East Nusa Tenggara, and South Sumatera provinces; were able to read and understood the Indonesian language and willing to participate in this study. Women with a history of breast cancer and who were pregnant or breastfeeding were excluded. A questionnaire was then administered from March to May 2016 to the eligible participants. Participants were collected until the data collection was completed by means of a participant self-report. Of the total 827 participants with a response rate of 94.8% successfully participated in this study.

*Data collection process*

The questionnaire used in this study was the BCAS-I that has been validated in Solikhah et al., (2017). The dependent variable of this study was breast cancer screening consisted of Yes-No questions that “did you ever have clinical breast examination (CBE) or mammogram or ultrasonography ever at any point per year in their life”. Confirming that they have done mammography/CBE/ultrasonography, the data from the interview results are then confirmed in the registration data at the primary health care centre, and if the participant is diagnosed with breast cancer, then it is categorized as “Yes” and vice versa. Independent variables were age, religion, education level, marital status, occupation, monthly income, health insurance, and knowledge of breast cancer risk factors, knowledge of signs, and symptoms. Knowledge was measured using a categorical response scale (yes, no, or don’t know) on possible symptoms of breast cancer and understanding the risk factors of breast cancer. Items with correct answers were given 1 point, while the incorrect answers received 0 point. Results were then classified as categorical data (poor knowledge versus good knowledge).

*Data analysis*

Data on participant characteristics were analyzed descriptively with means and standard deviation for continuous data and percentages for categorized data. The chi-square test was then performed to explore the relationship between sociodemographic, knowledge of breast cancer risk factors, signs, and symptoms, and breast cancer screening attendance. If the chi-square test assumptions were violated (e.g. the expected value was <5), the Fisher’s Exact test was employed. The binary logistic regression model was performed to estimate the odds ratio (OR) and 95% confidence intervals for the associations between sociodemographic characteristics, knowledge of breast cancer risk factors, signs, and symptoms, and breast cancer screening. Step-ward regression modelling was used to identify factors associated with breast cancer screening. Multicollinearity between independent variables was checked using STATA version 14 software package.

## Results

The average age of the participants in this study was 29.91 (± 11.14) years old with most of them were students (34.46%). The education level of the participants was distributed across the elementary school, junior high school, senior high school, undergraduate, and postgraduate with 8.71%, 7.13%, 38.57%, 43.05, and 2.54%, respectively. More than half of the participants had a monthly income of less than 2,000,000 IDR (<152 USD) (77.15%). Good knowledge of breast cancer risk factors, signs, and symptoms was seen in 69.77% (n=577) of the subjects (Table 1). Unsurprisingly, the majority of participants were not covered by health insurance. The prevalence of breast cancer screening was 18.74% (95% CI: 16.22–21.55) (Table 2). 

Results of the binary logistic regression model on the association between all independent variables and breast cancer screening are presented in Table 3. Christian women had a significantly lower breast cancer screening attendance compared to those from other religions (adj. OR. = 0.45, 95% CI: 0.24 – 0.85). Of all demographic variables, being an adult (35 – 39 years old) (adj. OR. = 1.52, 95% CI: 1.02 – 2.26) and having a household income of ≥6,000,000 IDR (≥457 USD) (adj. OR. = 5.19, 95% CI: 1.43 – 18.84) seemed to significantly increase the breast cancer screening attendance. Moreover, the odds of women with good knowledge of breast cancer to attend breast cancer screening was 1.75 higher to compared to those with poor knowledge (adj. OR = 1.75, 95% CI: 1.20 – 2.56).

**Table 1:** Baseline Characteristic of Participants (n= 827)

Characteristics	Number	Percentage (%)
State residency		
Yogyakarta	194	23.46
East Nusa Tenggara	263	31.88
South Sumatera	370	44.66
Age, years		
Early adulthood (18 – 34)	584	70.62
Adulthood (35 – 39)	222	26.84
Elderly (> 60)	21	2.54
Mean (±SD	29.91 (± 11.14)	
Median (min: max)	25 (18 : 80)	
Religion		
Muslim	547	66.14
Christian	50	6.05
Other	230	27.81
Education level		
Elementary school	72	8.71
Junior high school	59	7.13
Senior high school	319	38.57
Undergraduate	356	43.05
Postgraduate degree	21	2.54
Marital status		
Single	411	49.70
Married	384	46.43
Widowed/separated/discovered	32	3.87
Occupation		
Unemployed	149	18.02
Farmer	48	5.80
Student	285	34.46
Labourer	128	15.48
Government/official/enterprise/ business employees	217	34.46
Monthly income		
<2,000,000 IDR* (<152 USD)	638	77.15
2,000,000 – 6,000,000 IDR (152 – 457 USD)	180	21.77
≥6,000,000 IDR (≥457 USD)	9	1.09
Health insurance		
No	507	61.31
Yes	320	38.69
Knowledge of breast cancer risk factors, signs, and symptoms		
Poor	250	30.23
Good	577	69.77

*IDR, Indonesian rupiah rate; USD, United States Dollar

**Table 2:** Prevalence of Breast Cancer Screening among Women in Indonesia (n = 827)

Breast Cancer Screening	Number	Percentage	95%CI
Yes	155	18.74	16.22 – 21.55
No	672	81.26	78.45 – 21.55

**Table 3:** Odds Ratios and Adjusted Odds Ratios for Each Category of Factors Influencing Breast Cancer Screening Attendance (n = 827)

Characteristics	N	Breast cancer screening		Crude OR	95%CI	Adjusted Crude OR	95%CI
		No N (%)	Yes N (%)				
Age, years							
Early adulthood (18 – 34)	584	480 (82.19)	104 (17.81)	1		1	
Adulthood (35 – 39)	222	173 (77.93)	49 (22.07)	01.04	0.78 – 1.40	1.52*	1.02 – 2.26
Elderly (> 60)	21	19 (90.48)	2 (9.52)	0.36*	0.14 – 0.93	0.56	0.18 – 1.52
Religion							
Muslim	547	432 (78.98)	115 (21.02)	1		1	
Christian	50	43 (86.00)	7 (14.00)	0.55*	0.30 – 0.99	0.45*	0.24 – 0.85
Other	230	197 (85.65)	33 (14.35)	1.28	0.97 – 1.70	0.97	0.68 – 1.37
Education level							
Elementary school	72	58 (80.56)	14 (19.44)	1		1	
Junior high school	59	49 (83.05)	10 (16.95)	0.74	0.36 – 1.51	0.77	0.37 – 1.63
Senior high school	319	266 (83.39)	53 (16.61)	1.50	0.89 – 2.52	1.44	0.81 – 2.56
Undergraduate	356	281 (78.93)	75 (21.07)	1.95**	1.17 – 3.27	1.76	0.98 – 3.16
Postgraduate degree	21	18 (85.71)	3 (14.29)	1.74	0.70 – 4.35	1.41	0.53 – 3.74
Marital status							
Single	411	337 (82.00)	74 (18.00)	1		1	
Married	384	306 (79.69)	78 (20.31)	0.77	0.59 – 1.00	0.69	0.48 – 1.00
Widowed/separated/discovered	32	29 (90.62)	3 (9.38)	0.51	0.26 – 1.04	0.65	0.29 – 1.46
Occupation							
Unemployment	149	126 (84.56)	23 (15.44)	1		1	
Farmer	48	37 (77.08)	11 (22.92)	0.81	0.44 – 1.49	1.16	0.58 – 2.31
Student	285	231 (81.05)	54 (18.95)	0.66*	0.46 – 0.95	0.74	0.50 – 1.10
Labourer	128	107 (83.59)	21 (16.41)	0.60*	0.39 – 0.94	0.63	0.37 – 1.07
Government/official/enterprise/ business employees	217	171 (78.80)	46 (21.20)	0.76	0.52 – 1.11	0.70	0.42 – 1.17
Monthly income							
<2,000,000 IDR* (<152 USD**)	638	532 (83.39)	106 (16.61)	1		1	
2,000,000 – 6,000,000 IDR (152 – 457 USD)	180	135 (75.00)	45 (25.00)	1.38*	1.01 – 1.89	1.38	0.95 – 2.02
≥6,000,000 IDR (≥457 USD)	9	5 (55.56)	4 (44.44)	3.97*	1.15 – 13.76	5.19*	1.43 – 18.84
Health insurance							
No	507	262 (81.88)	58 (18.12)	1		1	
Yes	320	410 (80.87)	97 (19.13)	1.35*	1.03 – 1.77	1.30	0.98 – 1.72
Knowledge of breast cancer risk factors, signs, and symptoms							
Poor	250	188 (75.20)	62 (24.80)	1		1	
Good	577	484 (83.88)	93 (16.12)	0.72*	1.19 – 2.47	1.75*	1.20 – 2.56**

*p value < 0.050; ** p value <0.010; ***p value <0.001

## Discussion

This study sought to elucidate the determinants and its association with breast cancer screening attendance among women in Indonesia. At present, the specific cause of breast cancer has yet to be identified. Many women, especially in developing countries like Indonesia, are diagnosed with advanced breast cancer, which negatively impacts their survival rate (Jedy-Agba et al., 2016; Rivera-Franco and Leon-Rodriguez, 2018). Lack of knowledge of the early signs and symptoms of breast cancer and the poor awareness on the importance of early detection is the major cause of the late diagnosis among women, which is more apparent in countries with limited resources such as Indonesia (Jedy-Agba et al., 2016), particularly in countries with limited resources like in Indonesia. Breast cancer patients who are diagnosed in advanced stages have a poor prognosis and a low survival rate.

The present study revealed that knowledge of breast cancer is indeed associated with breast cancer screening attendance in Indonesian women. This finding confirms the association between knowledge of breast cancer risk, signs, and symptoms and breast cancer screening attendance observed in another population (Asadi et al., 2018; Dibisa et al., 2019). These may relate to the likelihood that women with relevant knowledge tend to make better decision to participate in breast cancer screening because they are able to weigh between the advantages and disadvantages of participating in the screening, thus able to make appropriate choices (Hersch et al., 2018). 

Furthermore, the result of this study demonstrated a low level of awareness towards breast cancer screening among Indonesian women. This poor awareness might be the consequences of inadequate education level and knowledge on the importance to do mammography or ultrasonography when there is a change in shape or bleeding in the breast. Inadequate household income also affects the decision of women to have early breast cancer screening earlier. The result of this study is in line with that of a previous study on Korean women that demonstrated poverty as affecting women’s ability to pay for cancer screening (Choi et al., 2018). 

Although systematic reviews have revealed that breast cancer screening might give negative effects for breast cancer patients such as false positive, false negative, over-diagnoses, pain during mammography-use, and psychological impact (Ende et al., 2017; Sankatsing et al., 2015; Stout et al., 2014), mammography, as the screening tool for early detection of breast cancer, has been shown to significantly save lives. About 5 in 10,000 women aged 40 - 49 years, 10 in 10,000 women aged 50 – 59 years and 42 out of 10,000 women aged 60 – 69 years received the benefits from having mammography (Baron et al., 2018). Breast cancer screening at ages 40 – 49 years is a proven strategy to reduce breast cancer deaths that it is suggested to start breast cancer screening at the age of 40 years old (Ray et al., 2017; Ohuchi et al., 2009). However, the results of a cost effectiveness analysis showed that screening is more effective in the 50-59 years age group compared to those under 50 years and over 60 years (Nguyen and Adang, 2018). A guideline for breast cancer screening in Columbia proposed that screening should be carried out routinely every 2 years under several strategies: annual screening for all women aged 50 to 74 years and annual screening for the risk group, which includes women with a family history of breast cancer diagnosis (Mar et al., 2018).

In addition to the education level, adult age (35-39 years old), Christian, and monthly income (the rich) affect the breast screening attendance positively. Age has been demonstrated as a factor that plays a role in a person’s behaviour in conducting breast health checks in another study (Abdel-Aziz et al., 2017). A previous study has shown that adults have a higher awareness towards the health of their breasts (Abolfotouh et al., 2015), which is supported by the finding in this study that adulthood is one of the influencing factors of the breast cancer screening (AdjOR. = 1.51; 95% CI: 1.01 – 2.24). Nonetheless, this is contrary to the finding in Saudi Arabia that presented the older age group to be more aware of having a breast check that the younger ones (Abdel-Aziz et al., 2017). Age is a key predictor in a person’s life experience that will positively associated with decision-making behaviour (Pratt et al., 2017). 

This study also showed that Christian women were significantly associated with lower breast cancer screening attendance. A small number of Christian breast cancer patients were identified in Indonesia (6.05%), which may reflect the fact that Christian is a minority religion in Indonesia, which is known as one of the countries with the biggest Muslim population. Most patients experience fear, anxiety, and discomfort when they are diagnosed for breast cancer and during the treatment, making it necessary for them to get support from family members, friend, and relatives. One of the form of coping distress among these patients is the stronger belief in God. Most Asian are influenced by culture, including kinship, and beliefs that family, significant others, and religion play a strong role in dealing with the critical phase of the disease (Lyu et al., 2019). 

Monthly income is also proven to have a strong influence on breast cancer screening in this study. This is in line with a previous study which explained that family finance relates to access to health (Jaffee et al., 2020). A study conducted in Singapore showed that the high cost of screening is perceived as a barrier to breast cancer screening (Malhotra et al., 2016). In addition, rich women had more flexibility to have health checks compared to those with low income. In contrast, poor women who have experience breast-health problems find it difficult to access health-services (Abolfotouh et al., 2015). A study that addressed barriers for breast cancer screening among women identified the financial problem, geographic and public transportation obstacles, long waiting times, physical obstacles (women with disabilities), inadequate healthcare provider skills and knowledge, and psychological distress of patients as the barriers for accessing the health service (George et al., 2018; Wells et al., 2017). Indicators for effective breast cancer screening, especially for developing countries like Indonesia, are affordable cost, painless methods, increased knowledge and awareness of breast cancer risk, good privacy, and shorter waiting time (Azami-Aghdash et al., 2015). The fact that women with a higher monthly (2,000,000 – 6,000,000 IDR) are significantly associated with breast cancer screening is our study support the findings in a previous study stating that limited financial and geographic concerns are perceived as barriers of breast cancer screening (Azami-Aghdash et al., 2015).

To the best our knowledge, this is the first large population-based study with a high response rate on breast cancer screening attendance. Despite the representativeness of the participants in terms of diverse cultures and geography of Indonesia, the education level of the participants, which is undergraduate education, does not represent the education level of the majority of Indonesian women. Nevertheless, the level of education is not shown to have a statistical association with breast cancer screening attitude that it can be deemed negligible. The limitation of this study originates from the fact that it is a single time-point study, resulting in a lack of information on the causal relationship between the risk factors for breast cancer screening. The self-reporting nature of this study can also introduce recall bias.

In conclusion, the overall breast cancer screening attendance is poor among women in Indonesia. Age, household income, religion, and knowledge of breast cancer risk factor were identified as factors that influence breast cancer screening attendance. Therefore, appropriate education on breast cancer should be provided. 

## Author Contribution Statement

All authors confirm contribution to the manuscript as follows: SS study conception, design, analysis data, LL and SS, analysed and interpreted data, RM and DSSR performed initial manuscript. All authors approved the final version of the manuscript. 
